# Supplementary material for: Toxicological Evaluation of SiO2 Nanoparticles by Zebrafish Embryo Toxicity Test
Source: Int J Mol Sci. 2019 Feb 18;20(4):882. doi: 10.3390/ijms20040882 (PMC6413002; doi:10.3390/ijms20040882)
Supplement: Supplementary file 1 [file ijms-20-00882-s001.pdf]

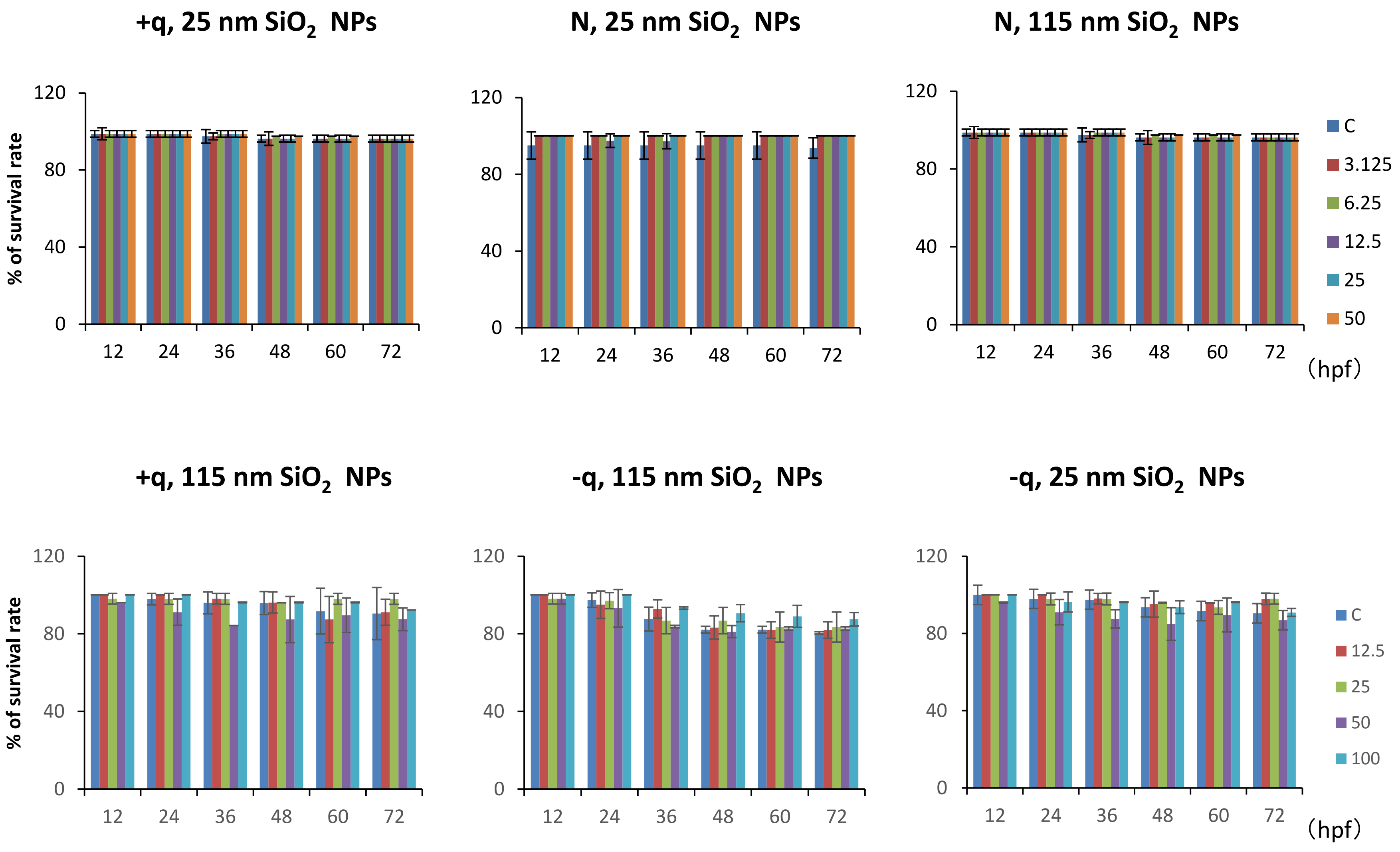

Figure S1. Survival rate at each time point of 12, 24, 36, 48, 60, and 72 hpf after exposure to 3.125, 6.25, 12.5, 25, 50, and 100 mg/L of 25- or 115-nm SiO_2 NPs with different surface charges. Data are represented as mean ± SD (standard deviation).

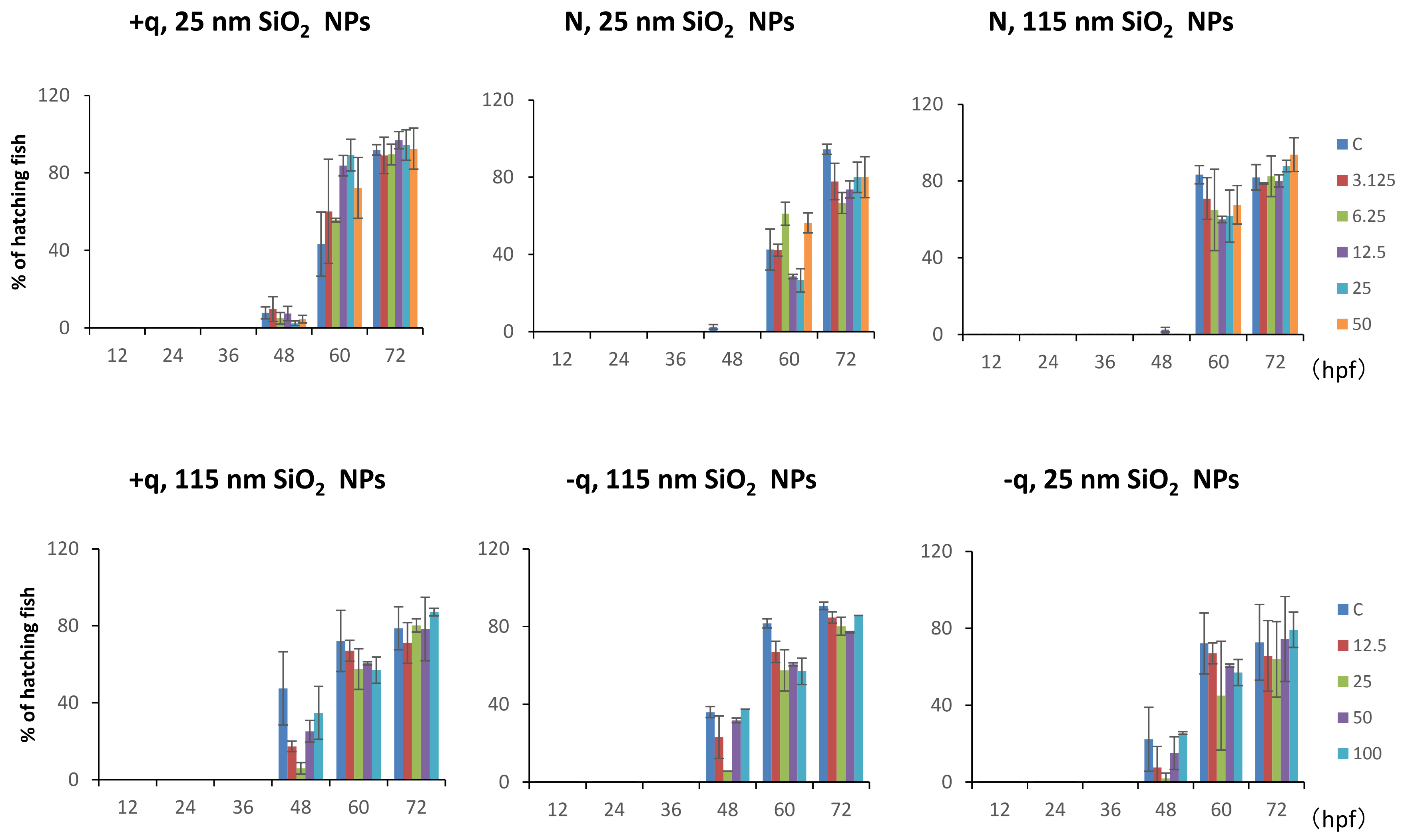

Figure S2. Hatching rate at each time point of 12, 24, 36, 48, 60, and 72 hpf after exposure to 3.125, 6.25, 12.5, 25, 50, and 100 mg/L of 25- or 115-nm SiO_2 NPs with different surface charges. Data are represented as mean ± SD (standard deviation).
